# Systemic inflammatory biomarkers are novel predictors of all-cause and cardiovascular mortality in individuals with osteoarthritis: a prospective cohort study using data from the NHANES

**DOI:** 10.1186/s12889-024-19105-5

**Published:** 2024-06-13

**Authors:** Erye Zhou, Jian Wu, Xin Zhou, Yufeng Yin

**Affiliations:** Department of Rheumatology and Immunology, The First Affiliated Hospital of Soochow University, No.188 Shizi St, Suzhou , Jiangsu, 215006 China

**Keywords:** Systemic inflammation, Biomarker, Mortality, Osteoarthritis

## Abstract

**Background:**

Chronic inflammation may contribute to increased mortality risk in individuals with osteoarthritis (OA), but research on the prognostic value of inflammatory biomarkers is limited. We aimed to evaluate the associations of the systemic immune–inflammation index (SII) and systemic inflammation response index (SIRI) with all-cause and cardiovascular mortality among US adults with OA.

**Methods:**

This cohort study included 3545 adults with OA aged ≥ 20 years from the National Health and Nutrition Examination Survey 1999–2020. The SII and SIRI were calculated using complete blood cell count data. Participants were categorized as having a higher or lower SII and SIRI using cutoff points derived by the maximally selected rank statistics method. Cox proportional hazards models, Fine–Gray competing risk regression models and time-dependent receiver operating characteristic (ROC) analysis were used to evaluate the associations between the SII/SIRI and mortality in OA patients.

**Results:**

Over a median follow-up of 5.08 (3.42–9.92) years, 636 (17.94%) deaths occurred, including 149 (4.20%) cardiovascular deaths. According to multivariable-adjusted models involving demographic, socioeconomic, and health factors, OA patients with a higher SII had a twofold greater risk of all-cause mortality than patients with a lower SII (HR 2.01; 95% CI: 1.50–2.68). Similarly, a higher SIRI was associated with an 86% increased risk of all-cause mortality relative to a lower SIRI (HR 1.86; 95% CI: 1.46–2.38). Similar to the trend found with all-cause mortality, patients with an elevated SII and SIRI had a 88% and 67% increased risk of cardiovascular mortality, respectively, compared to patients with a lower SII (HR 1.88; 95% CI: 1.16–3.03) and SIRI (HR 1.67; 95% CI: 1.14–2.44). Time-dependent ROC curves showed that both the SII and SIRI have moderate and valid performance in predicting short- and long-term mortality in patients with OA.

**Conclusions:**

Higher SII and SIRI values were associated with greater all-cause and cardiovascular mortality among US adults with OA.

**Supplementary Information:**

The online version contains supplementary material available at 10.1186/s12889-024-19105-5.

## Introduction

Osteoarthritis (OA) is the most common form of arthritis worldwide and a leading cause of chronic disability [1]. In the United States, OA affects approximately 30 million adults and results in substantial clinical and economic burdens [2]. OA is a heterogeneous disease characterized by the degeneration of articular cartilage and subchondral bone remodeling [3]. Although historically considered a form of noninflammatory arthritis, substantial evidence over the past decade recognizes chronic low-grade inflammation as a key factor in OA pathogenesis and progression [4]. The local production of inflammatory mediators within joint tissues directly contributes to cartilage breakdown and joint symptoms in OA [5, 6]. Moreover, systemic inflammation may increase OA severity through effects on muscle mass and function [7].

Despite major advances in OA research, no disease-modifying drugs are currently available, making management focused largely on symptom relief [8, 9]. Furthermore, while OA is rarely the direct cause of death, individuals with OA have a significantly greater risk of all-cause and cardiovascular mortality than does the general population [10, 11]. The mechanisms underlying this excess mortality are multifactorial but may be partly driven by chronic inflammation [12]. Higher levels of circulating inflammatory biomarkers, such as interleukin-6 (IL-6) and C-reactive protein (CRP), are associated with increased mortality in patients with OA [13, 14]. However, research on the prognostic utility of inflammatory markers in OA remains limited.

The systemic immune–inflammation index (SII) and systemic inflammation response index (SIRI) are novel composite biomarkers of systemic inflammation computed from routine complete blood count measurements. These indices integrate information from neutrophil, lymphocyte and platelet counts into simple ratios that reflect both proinflammatory and anti-inflammatory responses. They were originally developed to predict survival and cancer recurrence [15, 16]. Further studies indicated that elevated SII and SIRI values are prognostic indicators of poor clinical outcomes not only in cancers but also in numerous other diseases, including cardiovascular and cerebrovascular conditions [17–19]. Nonetheless, research into the associations of the SII and SIRI with mortality risk in OA patients is lacking.

In this context, our study represents an innovative investigation exploring the relationships of the novel integrated inflammatory markers SII and SIRI with mortality among OA patients. By evaluating and comparing the utility of the SII and SIRI for mortality risk stratification, our objective was to delineate the potential role of these easily obtainable biomarkers in predicting adverse outcomes in OA patients. By identifying the most prognostic inflammatory biomarkers, our findings could help drive the creation of better risk stratification approaches that incorporate inflammatory profiles to reduce mortality rates in people with OA.

## Methods

### Study population

The data source was the National Health and Nutrition Examination Survey (NHANES) 1999–2020, an ongoing program of studies assessing the health and nutritional status of adults and children in the United States. These studies are overseen by the National Center for Health Statistics (NCHS). The NHANES combines interviews, physical examinations, and laboratory tests using a complex, multistage probability sampling design to provide representative data on the US civilian noninstitutionalized population. All participants provided written informed consent, and the institutional review board of the NCHS approved all procedures. Comprehensive information about the NHANES study design and data is publicly available on the official website (https://cdc.gov/nchs/nhanes/).

This analysis included adult participants aged ≥ 20 years with OA between 1999 and March 2020. OA was defined based on an affirmative response to the question “Has a doctor or other health professional ever told you that you had arthritis?”, and participants could respond with either “Yes” or “No.” To further differentiate between types of arthritis, individuals with a positive response were asked to specify the type of arthritis, with response options including “Rheumatoid arthritis,” “Osteoarthritis,” “Psoriatic arthritis,” “Other,” “Refused,” and “Do not know.”

### Determination of mortality outcomes

The mortality status of participants in the NHANES database was obtained by accessing the NHANES public-use linked mortality files (LMFs), which contain death certificate records from the National Death Index (NDI) collected by the NCHS from various population surveys (https://www.cdc.gov/nchs/data-linkage/mortality.htm). These files enable linkage of mortality variables to participants collected in NHANES, using the unique person-level sequence number (SEQN) identifier. The most recent LMF file offers data on the duration, measured in months, from the date of survey participation to the event of death or until the censoring date of December 31, 2019. Total mortality included deaths from all causes. Cardiovascular mortality was defined as death due to diseases of the circulatory system based on the International Classification of Diseases, 10th Revision codes (I00-I09, I11, I13, I20-I51, and I60–I69) (Table S1) [20].

### Measurement of the SII and SIRI

Peripheral complete blood count (CBC) measured at the Mobile Examination Center (MEC) visit was used to calculate the SII and SIRI. The SII was defined as the platelet count × neutrophil count/lymphocyte count, while the SIRI was defined as the neutrophil count × monocyte count/lymphocyte count [15, 16]. There are no established cutoffs to define high vs. low SII and SIRI values. Therefore, we used the maximally selected rank statistics method (MSRSM) to determine the optimal threshold points for the SII and SIRI related to all-cause mortality. This statistical approach, with the core of the log-rank test, can locate the most significant spot in continuous variables where the difference in survival rates is the most pronounced. This approach is highly effective and intuitive for time-to-event outcomes, especially survival data [21]. This data-driven approach identified cutoff points of 978.25 for the SII and 1.50 for the SIRI (Fig. S1). Participants were classified into two groups based on these cutoff points.

### Assessment of covariates

The demographic covariates included age, sex (male/female), and race/ethnicity (non-Hispanic white, non-Hispanic black, Mexican American, other Hispanic, and other/multiracial). Socioeconomic status was evaluated using educational attainment (less than 9th grade, 9-11th grade, high school graduate, some college, college graduate and above) and the poverty–income ratio (< 1.29, 1.3–3.49, ≥ 3.5). Lifestyle and health factors comprised body mass index (BMI) categories of underweight (< 18.5 kg/m^2^), normal (18.5–24.9 kg/m^2^), overweight (25–29.9 kg/m^2^), or obese (≥ 30 kg/m^2^); smoking status (current smoker vs. nonsmoker); and health insurance status (insured vs. uninsured).

### Statistical analysis

Our analyses meticulously accounted for the complex NHANES survey design, incorporating sampling weights to ensure nationally representative estimates. Specifically, we utilized MEC exam weights (WTMEC4YR, WTMEC2YR, and WTMEPRP) in our analysis, enhancing the accuracy and validity of our results. The data were analyzed using R software version 4.3.2 (R Project for Statistical Computing, Vienna, Austria) with the ‘survival’, ‘maxstat’, ‘cmprsk’, ‘rms’ and ‘timeROC’ packages. The significance threshold was set at < 0.05 (two-tailed tests).

The baseline characteristics of the study population were summarized using means and standard deviations (SDs) for continuous variables and frequencies with unweighted percentages for categorical variables. Various statistical methods were employed based on the nature of the variables under investigation. Analysis of variance was used to assess differences in continuous variables among multiple groups, while the Wilcoxon rank-sum test was used to evaluate differences in ordered variables between two independent groups. For categorical variables, the chi-square test with Rao & Scott’s second-order correction was used, as it is designed to handle complex surveys or clustered data, thus ensuring accurate analysis in studies with complex samples.

We conducted univariate and multivariate analyses to assess the associations between mortality and systemic inflammatory biomarkers. In univariate analyses, the Kaplan‒Meier method along with log-rank tests were used to analyze the cumulative incidence of all-cause mortality between the higher and lower SII and SIRI groups. Univariate Fine–Gray competing-risk regression models were utilized to analyze the cumulative incidence of cardiovascular mortality while accounting for the competing risk of noncardiovascular mortality [22]. For the multivariate analyses, Cox proportional hazards models and multivariate Fine–Gray models were used to estimate hazard ratios (HRs) and 95% confidence intervals (CIs) for all-cause mortality and cardiovascular mortality, respectively. Three models were developed in these multivariate analyses: Model 1 was an unadjusted model. Model 2 was adjusted for demographic factors, including age, sex, race/ethnicity, and educational attainment. Model 3 made further adjustments for smoking status, BMI, health insurance status, and poverty–income ratio in addition to demographic factors.

Subgroup analyses were conducted based on prespecified effect modifiers (sex, age group, race/ethnicity, education, and other factors). In addition, to assess the robustness of the associations, a series of sensitivity analyses were separately performed after excluding certain groups of participants. A restricted cubic spline (RCS) with three knots was used to assess nonlinear dose‒response relationships between the SII/SIRI and mortality outcomes. Due to the right-skewed distribution of the original SII and SIRI values, natural logarithmic and square root transformations were applied for normalization. The ln-transformed SII and SIRI values exhibited adequate normality and were used in all RCS models (Fig. S2). Finally, time-dependent receiver operating characteristic (ROC) analyses were performed to assess the accuracy of the SII/SIRI in predicting survival outcomes at different time points (3, 5, and 10 years).

## Results

### Baseline characteristics

**Fig. 1:**
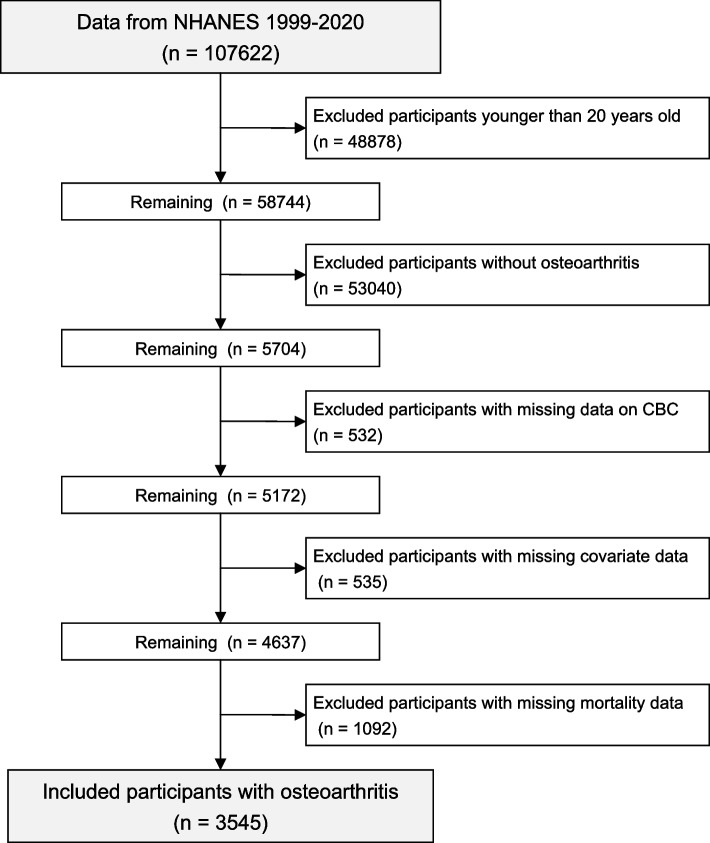
Flowchart of participant inclusion and exclusion criteria. Abbreviations: *NHANES*, National Health and Nutrition Examination Survey; *CBC*, complete blood count

The step-by-step process of participant selection for the analytical sample is illustrated in Fig. 1. Initially, a total of 107,622 individuals from the NHANES between 1999 and March 2020 were considered. Of the initial participants, 48,878 individuals under 20 years old were excluded. Additionally, 53,040 individuals without OA were also excluded. A further 532 and 535 participants were excluded due to missing CBC data and insufficient covariate data, respectively. After excluding 1092 participants with missing mortality data, the final study sample consisted of 3545 adults who were diagnosed with OA.

Among 3545 US adults with OA, the mean age was 61.22 years, and 65.65% were women. The study sample comprised non-Hispanic whites (85.16%), non-Hispanic blacks (5.70%), Mexican Americans (2.66%), and other Hispanics (2.14%). Approximately half of the participants (52.96%) were smokers, and 93.52% had health insurance. A total of 356 (10.04%) patients had a higher SII, and 1081 (30.49%) patients had a higher SIRI. Compared to participants with a lower SII, participants with a higher SII were older, had a greater waist circumference, and had a lower poverty–income ratio (all *p* < 0.05). Participants with a higher SIRI were also older but had higher educational attainment and poverty–income ratios than participants with a lower SIRI (both *p* < 0.05). Other characteristics were mostly similar between groups of individuals in the higher or lower classifications of the SII and SIRI (Table 1).

**Table 1:** Baseline characteristics of participants with osteoarthritis according to the SII and SIRI

Characteristic	Overall (*N* = 3545)	SII			SIRI		
		**Higher SII** **(*N* = 356)**	**Lower SII** **(*N* = 3189)**	***P* value**	**Higher SIRI** **(*N* = 1081)**	**Lower SIRI** **(*N* = 2464)**	***P* value**
No. (weighted)	36,055,832	3,534,313	32,521,519		10,880,025	25,175,807	
Age (years)	61.22 (13.31)	60.49 (14.93)	61.29 (13.12)	0.607	63.37 (13.87)	60.29 (12.96)	< 0.001
Age group				0.062			< 0.001
20–39 years	191 (5.85%)	25 (9.24%)	166 (5.48%)		53 (5.44%)	138 (6.03%)	
40–59 years	899 (32.57%)	93 (34.92%)	806 (32.32%)		221 (27.08%)	678 (34.95%)	
60–79 years	1760 (46.55%)	148 (40.42%)	1612 (47.22%)		513 (47.77%)	1247 (46.03%)	
≥ 80 years	695 (15.02%)	90 (15.42%)	605 (14.98%)		294 (19.71%)	401 (12.99%)	
Sex				0.744			< 0.001
Female	2261 (65.65%)	223 (66.63%)	2038 (65.54%)		577 (56.71%)	1684 (69.51%)	
Male	1284 (34.35%)	133 (33.37%)	1151 (34.46%)		504 (43.29%)	780 (30.49%)	
Race/ethnicity				0.052			< 0.001
Mexican American	312 (2.66%)	30 (2.80%)	282 (2.64%)		91 (2.61%)	221 (2.68%)	
Other Hispanic	191 (2.14%)	18 (2.64%)	173 (2.08%)		51 (1.99%)	140 (2.20%)	
Non-Hispanic White	2383 (85.16%)	274 (88.77%)	2109 (84.77%)		835 (89.65%)	1548 (83.22%)	
Non-Hispanic Black	488 (5.70%)	25 (3.38%)	463 (5.95%)		70 (2.67%)	418 (7.01%)	
Other/multiracial	171 (4.34%)	9 (2.41%)	162 (4.55%)		34 (3.07%)	137 (4.89%)	
Educational attainment				0.342			0.372
Less than 9th grade	310 (4.67%)	28 (4.79%)	282 (4.65%)		89 (5.04%)	221 (4.51%)	
9-11th grade	467 (10.37%)	48 (13.50%)	419 (10.03%)		149 (12.14%)	318 (9.61%)	
High school graduate	822 (22.67%)	81 (22.89%)	741 (22.65%)		247 (22.45%)	575 (22.77%)	
Some college	1073 (32.95%)	115 (34.19%)	958 (32.81%)		325 (31.59%)	748 (33.54%)	
College graduate or above	873 (29.34%)	84 (24.63%)	789 (29.85%)		271 (28.78%)	602 (29.58%)	
Poverty–income ratio	3.18 (1.59)	3.04 (1.55)	3.20 (1.59)	0.122	3.06 (1.56)	3.24 (1.60)	0.009
Poverty–income ratio group				0.014			0.016
< 1.29	884 (16.47%)	75 (15.35%)	809 (16.59%)		263 (17.05%)	621 (16.22%)	
1.30–3.49	1404 (37.09%)	172 (45.76%)	1232 (36.14%)		464 (40.87%)	940 (35.45%)	
≥ 3.50	1257 (46.45%)	109 (38.88%)	1148 (47.27%)		354 (42.07%)	903 (48.34%)	
BMI	30.35 (7.23)	30.98 (7.71)	30.29 (7.17)	0.159	30.67 (7.87)	30.22 (6.93)	0.599
BMI group				0.339			0.869
Underweight	37 (0.99%)	6 (1.61%)	31 (0.92%)		11 (1.17%)	26 (0.91%)	
Normal weight	733 (21.64%)	85 (21.44%)	648 (21.66%)		241 (21.96%)	492 (21.50%)	
Overweight	1176 (33.05%)	110 (29.47%)	1066 (33.44%)		360 (32.16%)	816 (33.43%)	
Obese	1599 (44.32%)	155 (47.49%)	1444 (43.98%)		469 (44.71%)	1130 (44.16%)	
Smoking status				0.005			0.011
Nonsmoker	1653 (47.04%)	137 (38.50%)	1516 (47.97%)		439 (43%)	1214 (48.79%)	
Smoker	1892 (52.96%)	219 (61.50%)	1673 (52.03%)		642 (57%)	1250 (51.21%)	
Health insurance				0.550			0.441
Insured	3288 (93.52%)	333 (92.65%)	2955 (93.62%)		1013 (92.97%)	2275 (93.76%)	
Uninsured	257 (6.48%)	23 (7.35%)	234 (6.38%)		68 (7.03%)	189 (6.24%)	

Continuous variables are expressed as the means with standard deviations (SDs), while categorical variables are shown as counts and percentages

*Abbreviations*: *SII* Systemic immune–inflammation index, *SIRI* Systemic inflammation response index, *BMI* Body mass index

### Associations of the SII and SIRI with mortality

**Fig. 2:**
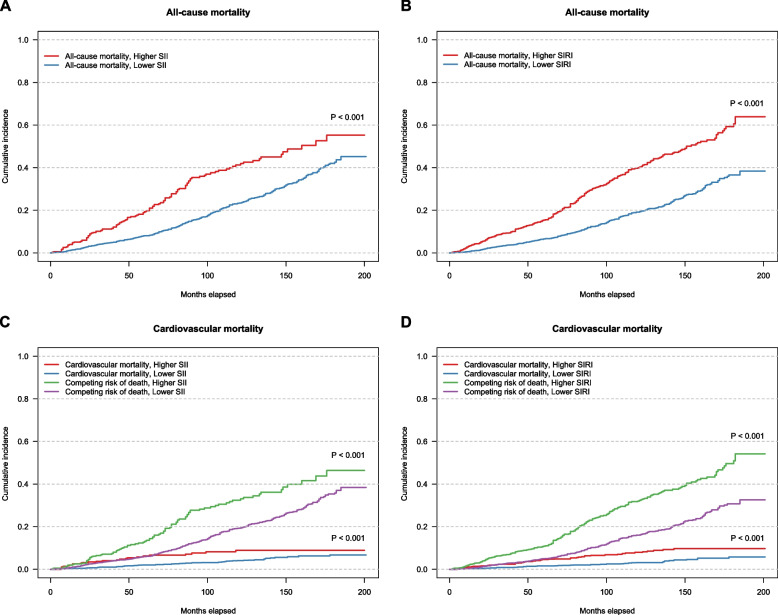
Cumulative incidence curves illustrating the effect of the SII/SIRI on mortality outcomes. **A**, **B**: Kaplan‒Meier curves for all-cause mortality stratified by the SII and SIRI. **C**, **D**: Competing risk analysis for cardiovascular mortality and competing risk of death for the SII and SIRI groups. The Y-axis indicates the cumulative incidence of mortality rate, starting from complete survival, across the months elapsed on the X-axis. Abbreviations: *OA*, osteoarthritis; *SII*, systemic immune–inflammation index; *SIRI*, systemic inflammation response index

During a median 5.08-year follow-up period (IQR 3.42–9.92 years), 636 all-cause deaths occurred, representing 17.94% of the cohort, along with 149 cardiovascular deaths, which accounted for 4.20% of the study sample. Figure 2 shows the Kaplan‒Meier curves for all-cause mortality and the univariate Fine–Gray models for cardiovascular mortality according to the SII and SIRI values among adults with OA. For all-cause mortality, the cumulative incidence of mortality was significantly greater in individuals with higher SII and SIRI values than in those with lower values (both *p* < 0.001). Individuals with a higher SII had approximately 41.9% more cumulative deaths at the 10-year follow-up than did those with a lower SII (23.4%). For the SIRI, the 10-year cumulative death rate was approximately 40.0% in the higher-SIRI group versus 19.3% in the lower SIRI group. Similarly, for cardiovascular mortality, higher SII and SIRI values were associated with substantially greater cumulative death probability over follow-up than lower SII and SIRI values (both *p* < 0.001).

Table 2 presents the associations between systemic inflammatory biomarkers and both all-cause and cardiovascular mortality, as revealed by multivariate analyses. According to the multivariate Cox models, participants in the higher SII subgroup had a significantly greater risk of all-cause mortality than did those in the lower SII subgroup (HR: 1.86, 95% CI: 1.39–2.49, *p* < 0.001) according to the unadjusted Model 1. This association remained significant after full adjustments in Model 3 (HR: 2.01, 95% CI: 1.50–2.68, *p* < 0.001). Consistent significant associations were also observed for the SIRI, with higher SIRIs associated with 2.43- and 1.86-fold increased risks of all-cause mortality in Models 1 and 3, respectively. For cardiovascular mortality, according to the multivariate Fine–Gray models, the higher-SII group showed a significantly increased risk in Model 1 (HR: 2.16, 95% CI: 1.38–3.40, *p* < 0.001), with a maintained association after full adjustment in Model 3 (HR: 1.88, 95% CI: 1.16–3.03, *p* = 0.010). The higher-SIRI group also demonstrated a significant association with cardiovascular mortality in the unadjusted and fully adjusted models (HR: 2.44, 95% CI: 1.71–3.50, *p* < 0.001 in Model 1 and HR: 1.67, 95% CI: 1.14–2.44, *p* = 0.009 in Model 3). In addition, the higher-SII and -SIRI groups had an increased subdistribution hazard for death due to competing risks due to noncardiovascular diseases.

**Table 2:** Hazard ratios of mortality according to the SII and SIRI among patients with osteoarthritis

Characteristic	Model 1		Model 2		Model 3	
	**HR (95% CI)**	***P* value**	**HR (95% CI)**	***P* value**	**HR (95% CI)**	***P* value**
**All-cause mortality**						
SII group	1.86 (1.39–2.49)	< 0.001	2.02 (1.53–2.67)	< 0.001	2.01 (1.50–2.68)	< 0.001
SIRI group	2.43 (1.94–3.05)	< 0.001	1.97 (1.57–2.48)	< 0.001	1.86 (1.46–2.38)	< 0.001
**Cardiovascular mortality**						
SII group	2.16 (1.38–3.40)	< 0.001	1.95 (1.21–3.15)	0.006	1.88 (1.16–3.03)	0.010
SIRI group	2.44 (1.71–3.50)	< 0.001	1.72 (1.17–2.53)	0.005	1.67 (1.14–2.44)	0.009
**Competing risk of death**						
SII group	1.82 (1.44–2.30)	< 0.001	1.77 (1.39–2.25)	< 0.001	1.73 (1.36–2.20)	< 0.001
SIRI group	2.20 (1.85–2.62)	< 0.001	1.81 (1.51–2.18)	< 0.001	1.77 (1.47–2.12)	< 0.001

Cox proportional hazards models were used to assess the risk of all-cause mortality based on the SII and SIRI. Multivariate Fine–Gray competing risk regression models were applied to evaluate the risk of mortality related to cardiovascular and noncardiovascular causes. Competing risks of death were identified as fatalities attributable to causes other than cardiovascular diseases. In these analyses, comparisons were made between groups with higher SII and SIRI values and those with lower SII and SIRI values. Model 1 was unadjusted. Model 2 was adjusted for age, sex, race/ethnicity, and educational attainment. Model 3 was adjusted for age, sex, race/ethnicity, educational attainment, smoking status, BMI, health insurance status, and the poverty–income ratio

*Abbreviations*: *SII* Systemic immune–inflammation index, *SIRI* Systemic inflammation response index, *BMI* Body mass index, *HR* Hazard ratio, *CI* Confidence interval

### Subgroup and sensitivity analyses

We found that the associations of a higher versus lower SII and SIRI with the risk of all-cause mortality were persistent across most subgroups, with a few exceptions. For the SII, the associations were not significant for participants aged 20–59 years, those who identified as “other/multiracial” race/ethnicity, those with educational attainment beyond high school, those who were uninsured, or those with a poverty–income ratio < 1.29 (all *p* values > 0.05). For the SIRI, the association with all-cause mortality was not significant among participants with educational attainment beyond high school and participants who were uninsured (all *p* values > 0.05) (Table 3).

**Table 3:** Subgroup analysis of the associations between the SII/SIRI and all-cause mortality among patients with osteoarthritis

Subgroup	SII		SIRI	
	**HR (95% CI)**	***P* value**	**HR (95% CI)**	***P* value**
Sex				
Male	2.11 (1.44–3.08)	< 0.001	1.58 (1.16–2.15)	0.003
Female	2.11 (1.47–3.02)	< 0.001	2.09 (1.56–2.80)	< 0.001
Age				
20–59 years	1.66 (0.74–3.73)	0.223	2.62 (1.43–4.81)	0.002
≥ 60 years	2.13 (1.58–2.88)	< 0.001	1.83 (1.41–2.37)	< 0.001
Race/ethnicity				
White	2.04 (1.48–2.81)	< 0.001	1.87 (1.44–2.42)	< 0.001
Other	1.88 (0.86–4.12)	0.113	2.52 (1.42–4.47)	0.002
Educational attainment				
High school or below	2.71 (1.80–4.07)	< 0.001	2.25 (1.58–3.19)	< 0.001
Beyond high school	1.46 (0.99–2.15)	0.059	1.62 (1.12–2.34)	0.010
BMI				
Non-obesity	1.98 (1.38–2.85)	< 0.001	1.98 (1.49–2.64)	< 0.001
Obesity	2.39 (1.51–3.77)	< 0.001	1.99 (1.37–2.89)	< 0.001
Smoking status				
Smoker	2.14 (1.51–3.03)	< 0.001	2.02 (1.48–2.76)	< 0.001
Nonsmoker	1.96 (1.21–3.19)	0.007	1.76 (1.19–2.60)	0.005
Health insurance				
Insured	2.02 (1.50–2.71)	< 0.001	1.90 (1.47–2.46)	< 0.001
Uninsured	3.74 (0.57–24.67)	0.171	3.62 (1.10–11.98)	0.035
Poverty–income ratio				
0–2.49	1.98 (1.39–2.80)	< 0.001	1.66 (1.23–2.23)	< 0.001
≥ 2.50	2.20 (1.51–3.19)	< 0.001	2.52 (1.82–3.48)	< 0.001

*Abbreviations SII* Systemic immune–inflammation index, *SIRI* Systemic inflammation response index, *BMI* Body mass index, *HR* Hazard ratio, *CI* Confidence interval

The associations between cardiovascular events and mortality based on the SII and SIRI were also generally consistent across subgroups. For the SII, the relationships were nonsignificant for participants who identified as non-Hispanic white race/ethnicity, those who were nonsmokers, those who were uninsured, and those with poverty–income ratios < 1.29 and ≥ 3.50 (all *p* > 0.05). For the SIRI, the associations with cardiovascular mortality were not significant in participants who were female, those who identified as “other/multiracial” race/ethnicity, those with an education beyond high school, nonsmokers, those who were uninsured, or those with poverty–income ratios of 0–2.49 (all *p* > 0.05) (Table 4).

**Table 4:** Subgroup analysis of the associations between the SII/SIRI and cardiovascular mortality in patients with osteoarthritis

Subgroup	SII		SIRI	
	**HR (95% CI)**	***P* value**	**HR (95% CI)**	***P* value**
Sex				
Male	2.06 (1.11–3.84)	0.022	2.06 (1.27–3.33)	0.003
Female	2.21 (1.10–4.42)	0.026	1.78 (1.00–3.19)	0.052
Age				
20–59 years	3.87 (1.07–13.98)	0.039	8.20 (2.17–31.03)	0.002
≥ 60 years	1.85 (1.12–3.07)	0.016	1.63 (1.11–2.40)	0.012
Race/ethnicity				
White	1.51 (0.90–2.52)	0.120	1.48 (0.99–2.21)	0.054
Other	5.48 (1.68–17.93)	0.005	2.21 (0.75–6.55)	0.150
Educational attainment				
High school or below	2.10 (1.05–4.20)	0.036	2.55 (1.46–4.46)	0.001
Beyond high school	1.99 (1.04–3.82)	0.037	1.56 (0.94–2.58)	0.086
BMI				
Obesity	2.52 (1.14–5.58)	0.023	2.01 (1.07–3.76)	0.030
Non-obesity	2.05 (1.16–3.62)	0.013	1.99 (1.26–3.14)	0.003
Smoking status				
Smoker	2.29 (1.32–3.96)	0.003	2.07 (1.30–3.28)	0.002
Nonsmoker	1.58 (0.63–3.96)	0.330	1.65 (0.86–3.18)	0.130
Health insurance				
Insured	2.02 (1.26–3.23)	0.003	1.91 (1.31–2.78)	0.001
Uninsured	1.68 (0.07–41.47)	0.750	0.55 (0.55–7.71)	0.657
Poverty–income ratio				
0–2.49	1.90 (1.04–3.45)	0.035	1.42 (0.88–2.30)	0.150
≥ 2.50	2.40 (1.15–5.02)	0.020	3.17 (1.72–5.85)	< 0.001

*Abbreviations*: *SII* Systemic immune–inflammation index, *SIRI* Systemic inflammation response index, *BMI* Body mass index, *HR* Hazard ratio, *CI* Confidence interval

The results of the sensitivity analyses are shown in Table S2-S5. According to the sensitivity analyses, similar findings were observed after excluding participants who died from cancer (*n* = 3415), those with other/unspecified causes of death (*n* = 3273), those younger than 40 years (*n* = 3335), or those with < 3 years of follow-up (*n* = 2851).

### Nonlinear relationships between the SII and SIRI and mortality

**Fig. 3:**
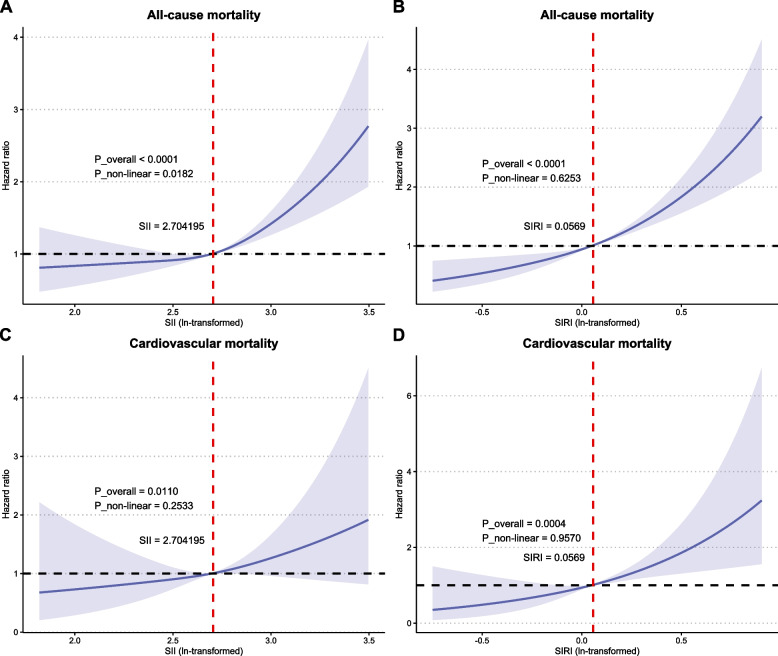
Restricted cubic spline fitting for the association between mortality and the SII and SIRI. **A**, **B**: all-cause mortality according to the SII and SIRI; **C**, **D**: cardiovascular mortality according to the SII and SIRI. The blue line indicates the HR, and the transparent area depicts the 95% CI. These analyses were adjusted according to Model 3. Abbreviations: SII, systemic immune–inflammation index; SIRI, systemic inflammation response index

RCS analyses revealed nuanced relationships between mortality outcomes and the SII and SIRI (Fig. 3). A nonlinear J-shaped association was observed between the SII and all-cause mortality risk (*p* overall < 0.001; *p* nonlinear = 0.0182), with risk progressively increasing with increasing SII values (Fig. 3A). In contrast, the SIRI exhibited a linear correlation with all-cause mortality (*p* overall < 0.001; *p* nonlinear = 0.6253) (Fig. 3B). For cardiovascular mortality, both the SII and the SIRI demonstrated a linear relationship (*p* overall < 0.05; *p* nonlinear > 0.05) (Fig. 3C, D).

### Predictive performance of the SII and SIRI for mortality

Figure S3 shows time-dependent ROC curves evaluating the prognostic performance of the SII/SIRI for predicting mortality at 3, 5, and 10 years. For all-cause mortality, the areas under the curve (AUCs) for the SII were 0.608, 0.557, and 0.549 at 3, 5, and 10 years, respectively. The AUCs for the SIRI were slightly greater at 0.658, 0.644, and 0.649 at 3, 5, and 10 years, respectively. For cardiovascular mortality, the SII had AUCs of 0.621, 0.563, and 0.528 at 3, 5, and 10 years, respectively. The SIRI again showed slightly greater prognostic accuracy, with AUCs of 0.716, 0.700, and 0.676 at 3, 5, and 10 years, respectively. Figure S4 illustrates the temporal trends in AUC values across different timepoints. These findings suggested that both the SII and the SIRI demonstrated valid and moderate performance for predicting all-cause and cardiovascular mortality that diminished slightly over longer time horizons.

## Discussion

With population aging, the incidence of OA is steadily increasing, making it a significant threat to human health. Numerous epidemiological studies have shown that immune responses and inflammation play pivotal roles in the development of OA [23]. To our knowledge, however, this is the first study to evaluate and demonstrate the prognostic utility of the SII and SIRI for mortality outcomes, specifically among individuals with OA. In this large nationally representative study of US adults, our results suggested that higher SII and SIRI values were associated with a significantly increased risk of all-cause and cardiovascular mortality among individuals with OA. These associations were independent of potential demographic, socioeconomic, and lifestyle confounders. Furthermore, our findings revealed a J-shaped nonlinear relationship between the SII and all-cause mortality, which aligns with previous results in the general population [24]. In summary, our findings suggest the clinical relevance of assessing chronic inflammation and support the likely contribution of systemic inflammatory pathways to excess mortality in OA patients.

OA is a prevalent degenerative joint disease and a leading cause of disability worldwide [25]. Although OA has traditionally been considered a noninflammatory form of arthritis, extensive evidence now demonstrates that chronic low-grade inflammation plays an integral role in OA onset and progression [26, 27]. Local inflammation within joint tissues promotes cartilage destruction and synovitis through effects on cartilage-degrading enzymes, apoptosis, and matrix proteins [28, 29]. Systematically, higher circulating levels of inflammatory mediators may accelerate OA severity through mechanisms including increased bone resorption and impaired muscle function [30, 31].

As systemic inflammatory biomarkers, the SII and SIRI have been linked to increased mortality rates in both the general population and individuals suffering from various diseases. In the general population, individuals with higher SII values showed a significant increase in all-cause mortality (HR 1.29) and cardiovascular mortality (HR 1.33) compared to those with lower SII values [32]. Similarly, adults with a higher SIRI also face a greater risk of all-cause and cardiovascular death than those with a lower SIRI [32]. In patients with congestive heart failure, another inflammatory condition, the SIRI and the SII were both significantly associated with mortality [33]. Notably, the SIRI demonstrated better prognostic discrimination for adverse clinical outcomes than did the CRP [33]. An elevated SII/SIRI has also been associated with increased risks of all-cause or cardiovascular mortality in individuals with obesity [34], nonalcoholic fatty liver disease [35], diabetes [36], and ischemic stroke [17]. Research on the utility of the SII and SIRI in OA has been limited, with very few studies examining their potential roles as biomarkers in this population. One study revealed that the incidence of knee OA (KOA) was 4.03-fold greater among individuals with an elevated SII than among those with a lower SII [37]. Another study reported that the SIRI (OR = 1.812) was an independent risk factor for OA disease activity [38]. However, these findings suggest that the SII and SIRI could be useful biomarkers for both the development and disease activity of OA. Our study builds on this preliminary evidence by demonstrating associations of elevated SII and SIRI with increased all-cause and cardiovascular mortality among OA patients. The observed relationships between a heightened SII/SIRI and a greater risk of mortality expand our understanding of their potential utility as biomarkers in this population.

The composite inflammatory indices SII and SIRI integrate circulating counts of platelets, neutrophils, monocytes, and lymphocytes, which collectively reflect pathways of thrombogenesis, inflammation, and adaptive immunity [39]. Platelets were historically viewed as fundamental elements in hemostasis; however, there is increasing awareness that they also play a vital role in both inflammatory and immune responses [40, 41]. Activated platelet release exacerbates the overall immune reaction and cartilage and bone degeneration in the affected area in OA [42]. Monocytes enter synovial tissue and differentiate into macrophages that secrete a host of inflammatory mediators that fuel OA progression [43]. A greater number of circulating neutrophils and monocytes signifies greater accumulation and inflammatory activity of these innate immune cells within joints in individuals with arthritis, including rheumatoid OA [44–46]. A decrease in lymphocytes, observed in the synovial membrane of advanced OA patients, leads to impaired immune function and immunological dysfunction [32, 47]. Compared to individual cell counts, the composite indices SII and SIRI have several advantages in terms of their predictive ability. Single blood cell measures can be readily confounded by factors influencing cell volume, whereas the ratios of the SII and SIRI enhance stability. Additionally, integrating three distinct inflammatory cell types captures their interdependent effects and cumulative impact. This might provide a more robust reflection of the overall inflammatory status.

In our study, the associations between the SII and SIRI and mortality outcomes in individuals with OA were not entirely homogeneous across subgroups. For all-cause mortality, the association with an elevated SII was most pronounced in older adults older than 60 years. This divergent age effect implies that the SII may capture different aspects of immune dysfunction affecting mortality risk. Aging is characterized by chronic, low-grade inflammation that typically leads to tissue damage or degeneration [48]. This age-related inflammatory state is closely associated with morbidity and mortality [39]. Additionally, stronger associations were found between elevated SII and all-cause mortality in subgroups with lower education, obesity, and smoking history. Individuals with less education often face socioeconomic disadvantages and psychosocial stress linked to chronic low-grade inflammation [49]. Moreover, excessive adiposity drives inflammatory cytokine production and oxidative stress [50]. Smoking also provokes inflammatory responses and immune activation [50]. The proinflammatory effects of these factors likely interact with SII-associated immune impairment to further endanger health.

For cardiovascular mortality, the elevated SII and SIRI showed no significant differences across sex, age groups, education levels, or BMI categories. On the other hand, racial/ethnic minorities and smokers exhibited greater risk associated with increased SII and SIRI than did non-Hispanic white individuals and nonsmokers, respectively. The heightened cardiovascular mortality risk associated with elevated SII and SIRI in racial/ethnic minorities may stem from social and economic disparities faced by these groups [51]. The link between smoking and OA progression is likely complex, as nicotine may exert dichotomous effects [52]. However, the connection between smoking and mortality has been well established [53]. Although elevated SII and SIRI were associated with increased cardiovascular mortality in OA patients who smoked in our study, the exact interplay between systemic inflammation, smoking and OA outcomes warrants further investigation.

Several implications emerge from this study. First, a major strength of this study is the large, nationally representative sample of US adults followed longitudinally over an extensive period, with control for most potential confounding factors. Second, our results first highlight the potential value of the SII and SIRI as prognostic biomarkers of mortality risk in OA patients. These novel indices can be easily calculated from routine CBC without additional costs. Measurements of the SII and SIRI may help clinicians identify OA patients with the highest risk of mortality who may benefit from targeted risk reduction interventions. Third, we conducted multiple subgroup and sensitivity analyses to confirm the robustness of the observed associations.

Nonetheless, some limitations should be acknowledged. First, the single assessment of the SII and SIRI at baseline precluded evaluating changes over time. Second, the diagnosis of OA was based on patients’ self-reports, which may have led to bias. Nonetheless, a previous study demonstrated an 81% concordance rate between self-reported OA and clinically well-defined OA, indicating that in most cases, individuals are able to self-report OA with a high degree of accuracy [54, 55]. Third, while we controlled for most potential confounders, residual confounding from unmeasured factors cannot be excluded. Certain dietary patterns, genetic susceptibility, undiagnosed conditions, and psychological stress can modulate inflammation and vascular health but were unavailable for adjustment in our study. Fourth, our study lacked some data on participants’ acute inflammatory status and medication at the time of blood collection. For example, the use of steroids, nonsteroidal anti-inflammatory drugs, antibiotics, and their therapeutic durations were not documented. These factors could plausibly impact baseline leukocyte counts. Fifth, there was a strong correlation (Spearman’s *ρ* = 0.734, *p* < 0.001) between the SII and SIRI because of their shared reliance on neutrophil and lymphocyte counts in their formulations. This interdependency precludes their concurrent use as prognostic indicators for mortality in the same patient. Regrettably, no consensus has been reached on the interpretation of discordant prognostic information yielded by the SII and SIRI for the same patient. Thus, future studies should validate our findings in other cohorts, elucidate the underlying mechanisms, and determine whether lowering the SII and SIRI can reduce mortality in OA patients.

## Conclusion

In conclusion, higher SII and SIRI values, reflecting increased systemic inflammation, were independently associated with an increased risk of all-cause and cardiovascular mortality among US adults with OA. These widely available and inexpensive blood indices may serve as useful prognostic markers for identifying high-risk OA patients. Nonetheless, further research is warranted to elucidate the complex interrelationships between circulating inflammatory biomarkers and survival outcomes in individuals with OA.

### Supplementary Information

Supplementary Material 1.

## Data Availability

The datasets generated and analyzed in the current study are available at the NHANES website: https://www.cdc.gov/nchs/nhanes/index.htm.

## Abbreviations

AUC: Area under the curve
BMI: Body mass index
CDC: Center for Disease Control and Prevention
CRP: C-reactive protein
LMF: Linked mortality file
MSRSM: Maximally selected rank statistics method
NCHS: National Center for Health Statistics
NHANES: National Health and Nutrition Examination Survey
OA: Osteoarthritis
RCS: Restricted cubic splines
ROC: Receiver–operator characteristic curve
SII: Systemic immune–inflammation index
SIRI: Systemic inflammation response index
