# Supplementary material for: Systemic inflammatory biomarkers are novel predictors of all-cause and cardiovascular mortality in individuals with osteoarthritis: a prospective cohort study using data from the NHANES
Source: BMC Public Health. 2024 Jun 13;24:1586. doi: 10.1186/s12889-024-19105-5 (PMC11170786; doi:10.1186/s12889-024-19105-5)
Supplement: Supplementary file 1 — Supplementary Material 1. [file 12889_2024_19105_MOESM1_ESM.docx]

# Supplementary Material

# Systemic inflammatory biomarkers are novel predictors of all-cause mortality in individuals with osteoarthritis: A prospective cohort study using data from the NHANES

## Table S1 Codes for Leading Causes of Death in NHANES

| **Event in this study** | **UCOD_LEADING Recode** | **UCOD_113 Recode** | **Cause Title and** **ICD-10 Codes Included** |
| --- | --- | --- | --- |
| 120 | 001 | 054 - 064 | Diseases of heart (I00-I09, I11, I13, I20-I51) |
| 130 | 002 | 019 - 043 | Malignant neoplasms (C00-C97) |
| 26 | 003 | 082 - 086 | Chronic lower respiratory diseases (J40-J47) |
| 11 | 004 | 112 - 123 | Accidents (unintentional injuries) (V01-X59, Y85-Y86) |
| 29 | 005 | 070 | Cerebrovascular diseases (I60-I69) |
| 18 | 006 | 052 | Alzheimer’s disease (G30) |
| 15 | 007 | 046 | Diabetes mellitus (E10-E14) |
| 9 | 008 | 076 - 078 | Influenza and pneumonia (J09-J18) |
| 6 | 009 | 097 - 101 | Nephritis, nephrotic syndrome, and nephrosis (N00-N07, N17-N19, N25-N27) |
| 272 | 010 |  | All other causes (residual) |

Abbreviations: *LMF* linked mortality file; *UCOD_LEADING* the leading causes of death; *UCOD_113* the Underlying Cause of Death 113; *ICD–10* the International Classification of Diseases, 10th Revision.

The NHANES public-use linked mortality file (LMF) includes the UCOD_LEADING codes (001-010), which are derived from the more detailed UCOD_113 variable found in the restricted-use LMF. While the UCOD_113 codes categorize the underlying cause of death according to the ICD-10 standards


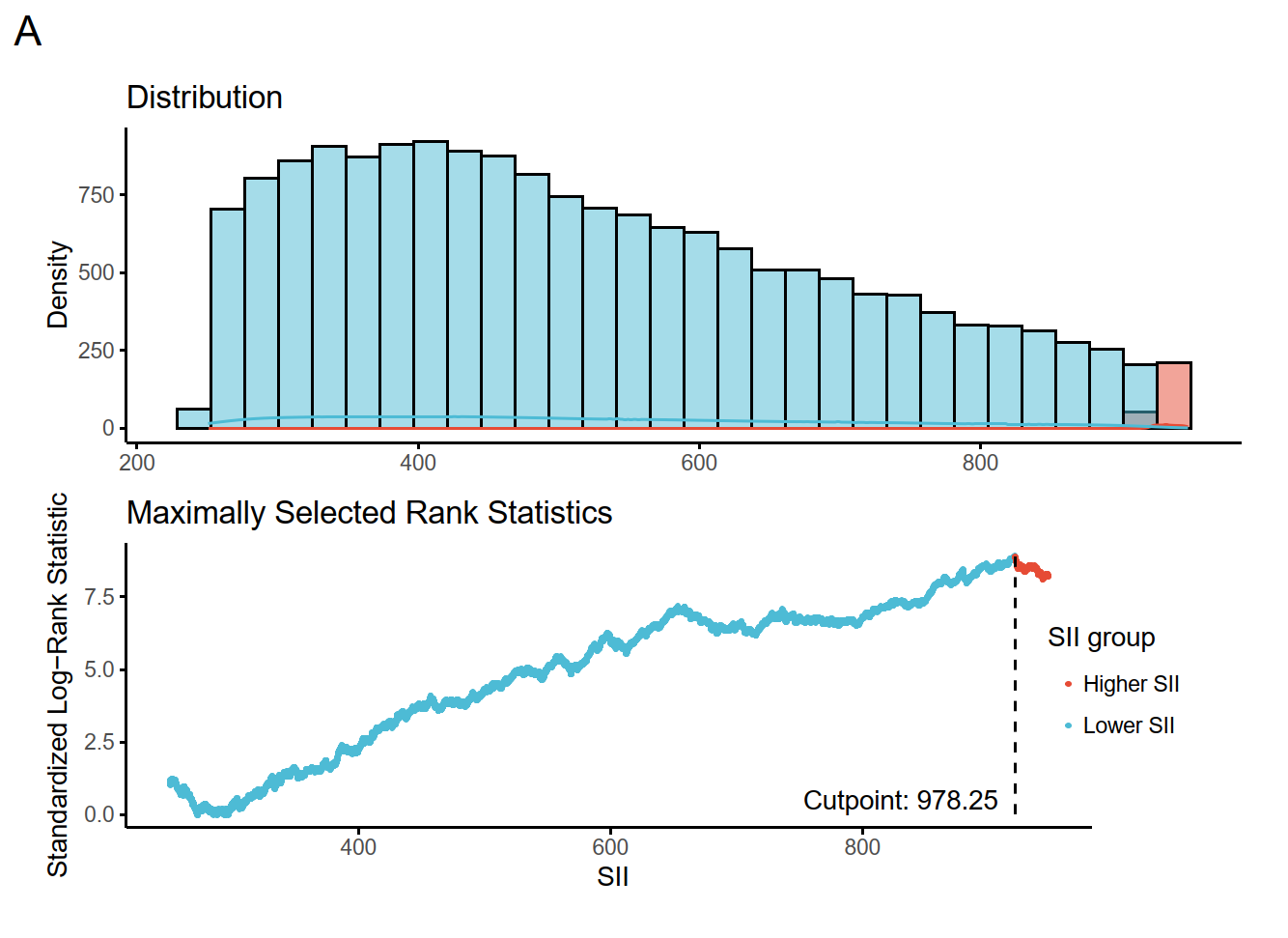

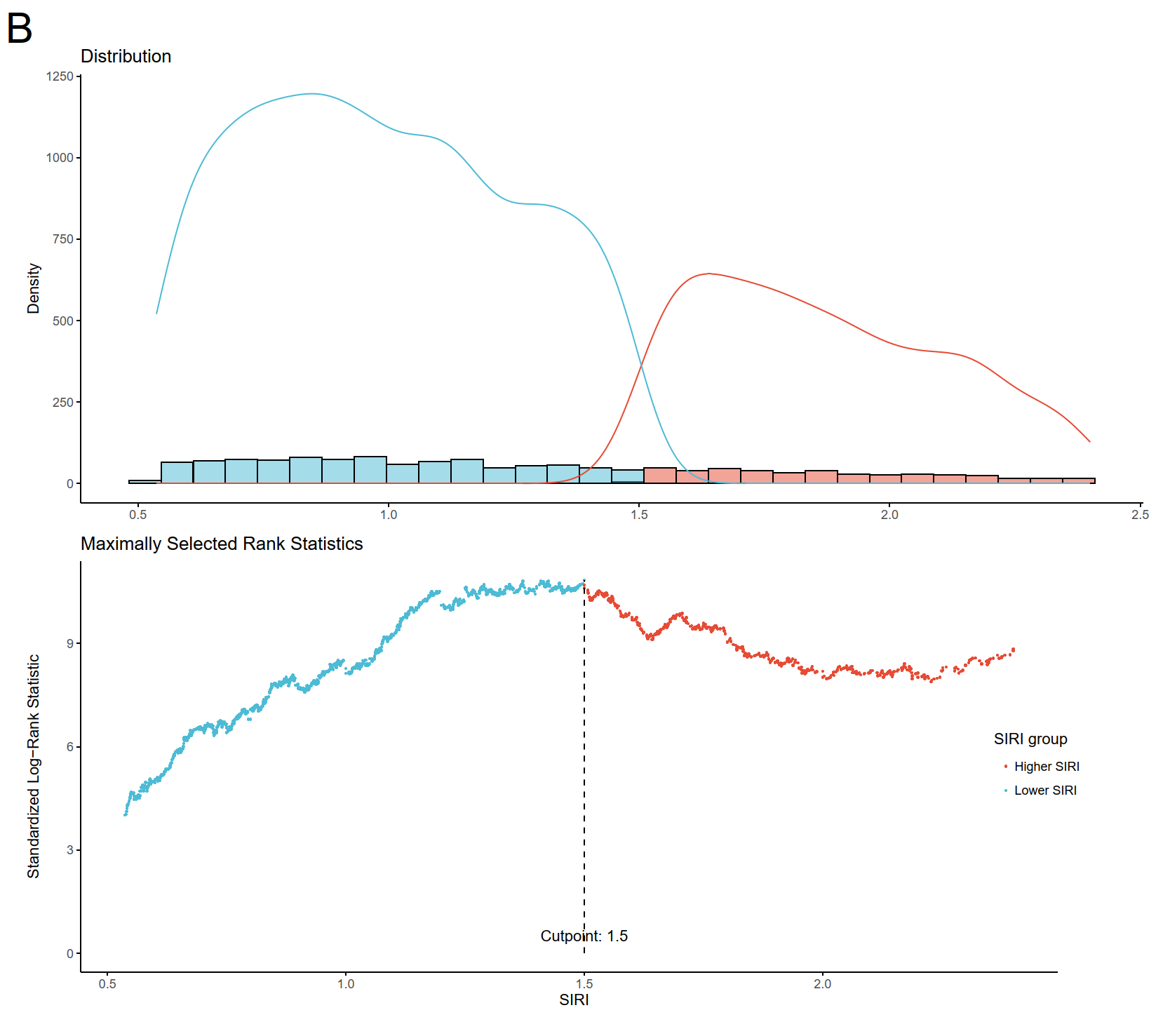


## Fig. S1 The optimal cutoff points of the SII (A) and SIRI (B) were calculated using MSRSM.

Abbreviations: *SII* systemic immune-inflammation index, *SIRI* systemic inflammation response index, *MSRSM* maximally selected rank statistics method.

The vertical dotted line indicates the optimal cut-off that maximizes the standardized log-rank statistic


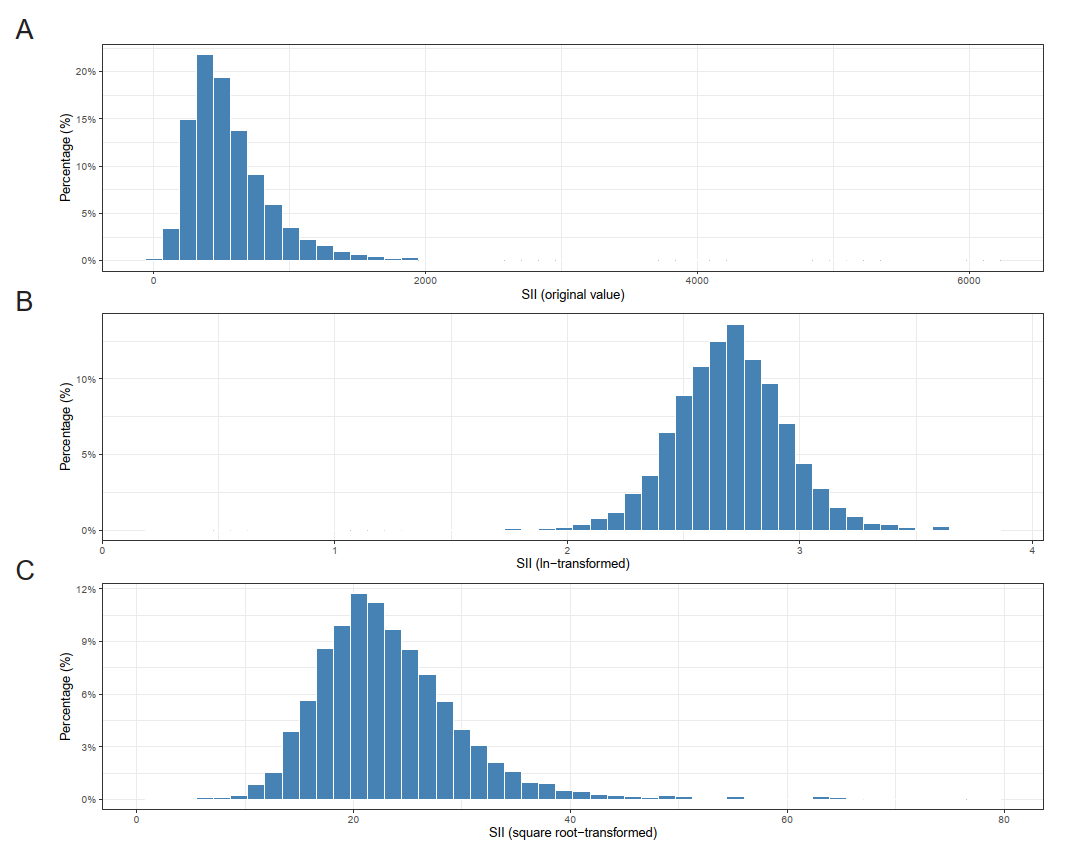

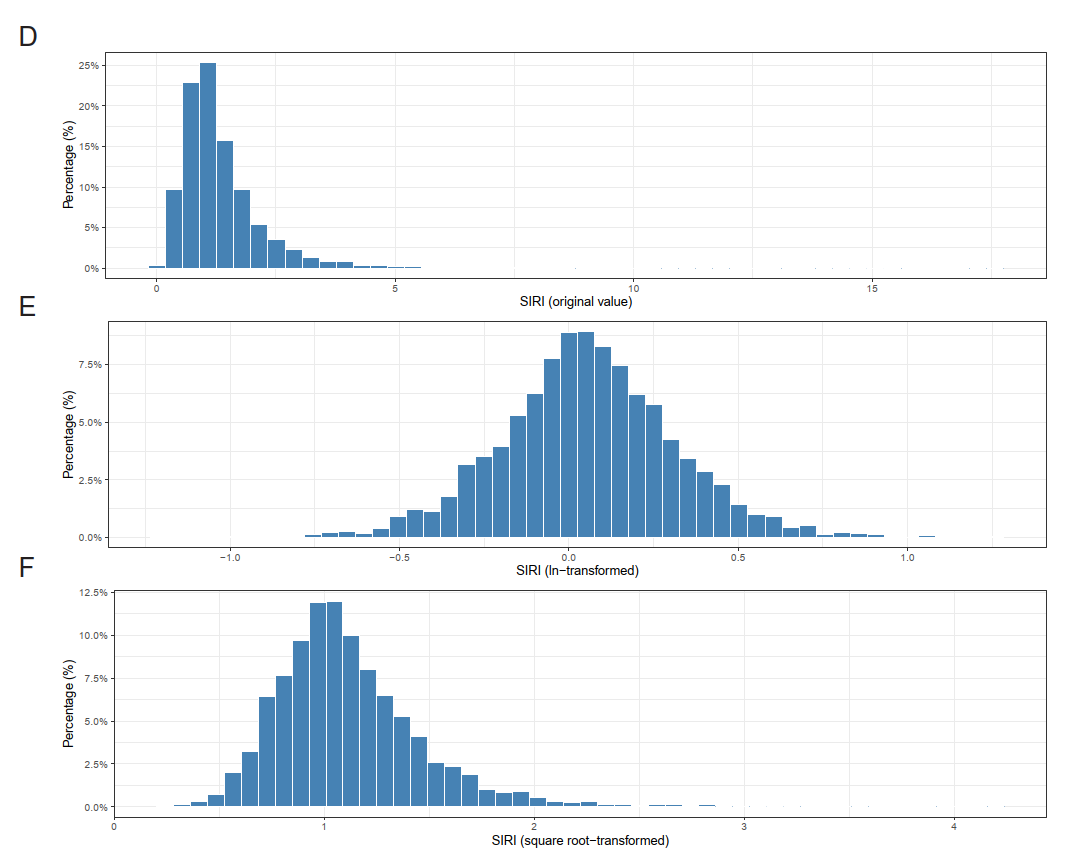


## Fig. S2 The distribution of the SII and SIRI of the original values (A, D), natural-logarithm (ln) converted values (B, E), and square-root converted values (C, F).

Abbreviations: *SII* systemic immune-inflammation index, *SIRI* systemic inflammation response index

## Table S2 HR (95% CIs) for mortality after excluding any participants who died from cancer (n = 3415).

| **Characteristic** | **Model 1** | |  | **Model 2** | |  | **Model 3** | |
| --- | --- | --- | --- | --- | --- | --- | --- | --- |
|  | **HR (95% CI)** | **P value** |  | **HR (95% CI)** | **P value** |  | **HR (95% CI)** | **P value** |
| **All-cause mortality** |  |  |  |  |  |  |  |  |
| SII group | 1.71 (1.30-2.26) | <0.001 |  | 1.64 (1.26-2.14) | <0.001 |  | 1.60 (1.21-2.11) | <0.001 |
| SIRI group | 2.59 (2.03-3.30) | <0.001 |  | 2.01 (1.57-2.58) | <0.001 |  | 1.90 (1.47-2.46) | <0.001 |
| **Cardiovascular mortality** |  |  |  |  |  |  |  |  |
| SII group | 2.21 (1.41-3.48) | 0.001 |  | 2.04 (1.26-3.30) | 0.004 |  | 1.95 (1.20-3.16) | 0.007 |
| SIRI group | 2.54 (1.78-3.64) | <0.001 |  | 1.76 (1.19-2.59) | 0.004 |  | 1.70 (1.15-2.50) | 0.007 |
| **Competing risk of death** |  |  |  |  |  |  |  |  |
| SII group | 1.88 (1.44-2.45) | <0.001 |  | 1.81 (1.38-2.38) | <0.001 |  | 1.78 (1.35-2.34) | <0.001 |
| SIRI group | 2.26 (1.85-2.75) | <0.001 |  | 1.80 (1.46-2.22) | <0.001 |  | 1.76 (1.43-2.17) | <0.001 |

The Cox proportional hazards models were employed to assess the risk of all-cause mortality. The Fine-Gray competing risk regression models were applied to evaluate the risk of cardiovascular. In these analyses, comparisons were made between groups with higher SII and SIRI values against those with lower SII and SIRI. Model 1 was unadjusted. Model 2 was adjusted for age, sex, race/ethnicity, and educational attainment. Model 3 was adjusted for age, sex, race/ethnicity, educational attainment, smoking status, BMI, health insurance, and the poverty–income ratio.

Abbreviations: *SII* systemic immune-inflammation index, *SIRI* systemic inflammation response index, *BMI* body mass index, *HR* hazard ratios, *CI* confidence interval.

## Table S3 HR (95% CIs) for mortality after excluding participants with other/unspecified causes of death (n = 3273)

| **Characteristic** | **Model 1** | |  | **Model 2** | |  | **Model 3** | |
| --- | --- | --- | --- | --- | --- | --- | --- | --- |
|  | **HR (95% CI)** | **P value** |  | **HR (95% CI)** | **P value** |  | **HR (95% CI)** | **P value** |
| **All-cause mortality** |  |  |  |  |  |  |  |  |
| SII group | 1.89 (1.33-2.70) | <0.001 |  | 2.20 (1.56-3.12) | <0.001 |  | 2.09 (1.45-3.02) | <0.001 |
| SIRI group | 2.41 (1.80-3.24) | <0.001 |  | 2.06 (1.52-2.78) | <0.001 |  | 2.03 (1.49-2.76) | <0.001 |
| **Cardiovascular mortality** |  |  |  |  |  |  |  |  |
| SII group | 2.37 (1.51-3.71) | <0.001 |  | 2.34 (1.44-3.80) | 0.001 |  | 2.23 (1.37-3.63) | 0.001 |
| SIRI group | 2.74 (1.92-3.93) | <0.001 |  | 1.97 (1.35-2.88) | <0.001 |  | 1.92 (1.32-2.81) | 0.001 |
| **Competing risk of death** |  |  |  |  |  |  |  |  |
| SII group | 1.83 (1.30-2.58) | <0.001 |  | 1.91 (1.34-2.71) | <0.001 |  | 1.82 (1.28-2.59) | 0.001 |
| SIRI group | 2.09 (1.63-2.70) | <0.001 |  | 1.82 (1.39-2.39) | <0.001 |  | 1.76 (1.34-2.32) | <0.001 |

The Cox models and Fine-Gray models were applied to evaluate the risk of all-cause and cardiovascular, respectively. In these analyses, comparisons were made between groups with higher SII and SIRI values against those with lower SII and SIRI. Model 1 was unadjusted. Model 2 was adjusted for age, sex, race/ethnicity, and educational attainment. Model 3 was adjusted for age, sex, race/ethnicity, educational attainment, smoking status, BMI, health insurance, and the poverty–income ratio.

Abbreviations: *SII* systemic immune-inflammation index, *SIRI* systemic inflammation response index, *BMI* body mass index, *HR* hazard ratios, *CI* confidence interval.

## Table S4 HR (95% CIs) for mortality after excluding participants younger than 40 years (n = 3335)

| **Characteristic** | **Model 1** | |  | **Model 2** | |  | **Model 3** | |
| --- | --- | --- | --- | --- | --- | --- | --- | --- |
|  | **HR (95% CI)** | **P value** |  | **HR (95% CI)** | **P value** |  | **HR (95% CI)** | **P value** |
| **All-cause mortality** |  |  |  |  |  |  |  |  |
| SII group | 1.99 (1.48-2.67) | <0.001 |  | 2.00 (1.51-2.65) | <0.001 |  | 2.01 (1.49-2.68) | <0.001 |
| SIRI group | 2.49 (1.97-3.14) | <0.001 |  | 1.98 (1.57-2.49) | <0.001 |  | 1.87 (1.46-2.40) | <0.001 |
| **Cardiovascular mortality** |  |  |  |  |  |  |  |  |
| SII group | 2.14 (1.35-3.38) | 0.001 |  | 1.84 (1.14-2.99) | 0.013 |  | 1.78 (1.10-2.90) | 0.020 |
| SIRI group | 2.40 (1.67-3.43) | <0.001 |  | 1.65 (1.12-2.42) | 0.011 |  | 1.60 (1.10-2.35) | 0.015 |
| **Competing risk of death** |  |  |  |  |  |  |  |  |
| SII group | 1.93 (1.52-2.43) | <0.001 |  | 1.78 (1.40-2.26) | <0.001 |  | 1.74 (1.37-2.22) | <0.001 |
| SIRI group | 2.25 (1.89-2.67) | <0.001 |  | 1.83 (1.52-2.20) | <0.001 |  | 1.79 (1.49-2.16) | <0.001 |

The Cox models and Fine-Gray models were applied to evaluate the risk of all-cause and cardiovascular, respectively. In these analyses, comparisons were made between groups with higher SII and SIRI values against those with lower SII and SIRI. Model 1 was unadjusted. Model 2 was adjusted for age, sex, race/ethnicity, and educational attainment. Model 3 was adjusted for age, sex, race/ethnicity, educational attainment, smoking status, BMI, health insurance, and the poverty–income ratio.

Abbreviations: *SII* systemic immune-inflammation index, *SIRI* systemic inflammation response index, *BMI* body mass index, *HR* hazard ratios, *CI* confidence interval.

## Table S5 HR (95% CIs) for mortality after excluding participants with <3 years of follow-up (n = 2851)

| **Characteristic** | **Model 1** | |  | **Model 2** | |  | **Model 3** | |
| --- | --- | --- | --- | --- | --- | --- | --- | --- |
|  | **HR (95% CI)** | **P value** |  | **HR (95% CI)** | **P value** |  | **HR (95% CI)** | **P value** |
| **All-cause mortality** |  |  |  |  |  |  |  |  |
| SII group | 1.47 (1.13-1.92) | 0.004 |  | 1.48 (1.15-1.89) | 0.002 |  | 1.45 (1.11-1.89) | 0.006 |
| SIRI group | 2.24 (1.71-2.94) | <0.001 |  | 1.81 (1.41-2.32) | <0.001 |  | 1.74 (1.35-2.24) | <0.001 |
| **Cardiovascular mortality** |  |  |  |  |  |  |  |  |
| SII group | 1.36 (0.71-2.60) | 0.350 |  | 1.19 (0.60-2.34) | 0.620 |  | 1.13 (0.57-2.23) | 0.730 |
| SIRI group | 2.26 (1.44-3.56) | 0.000 |  | 1.62 (1.01-2.59) | 0.047 |  | 1.56 (0.97-2.50) | 0.065 |
| **Competing risk of death** |  |  |  |  |  |  |  |  |
| SII group | 1.81 (1.37-2.38) | <0.001 |  | 1.79 (1.35-2.37) | <0.001 |  | 1.73 (1.30-2.30) | <0.001 |
| SIRI group | 2.10 (1.72-2.57) | <0.001 |  | 1.73 (1.40-2.13) | <0.001 |  | 1.68 (1.36-2.07) | <0.001 |

The Cox models and Fine-Gray models were applied to evaluate the risk of all-cause and cardiovascular, respectively. In these analyses, comparisons were made between groups with higher SII and SIRI values against those with lower SII and SIRI. Model 1 was unadjusted. Model 2 was adjusted for age, sex, race/ethnicity, and educational attainment. Model 3 was adjusted for age, sex, race/ethnicity, educational attainment, smoking status, BMI, health insurance, and the poverty–income ratio.

Abbreviations: *SII* systemic immune-inflammation index, *SIRI* systemic inflammation response index, *BMI* body mass index, *HR* hazard ratios, *CI* confidence interval.


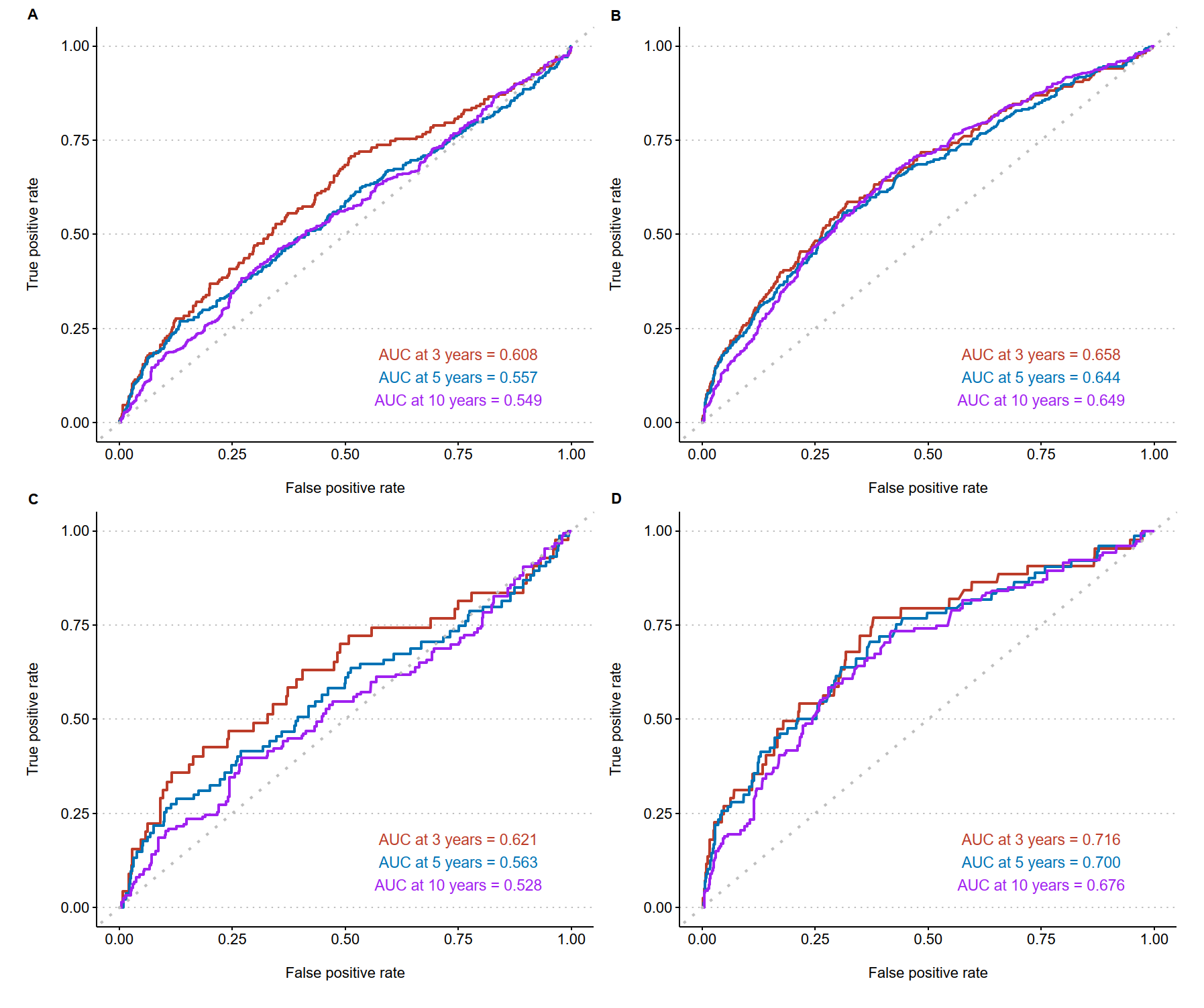


## Fig. S3 Time-dependent ROC curves for predicting survival at 3, 5, and 10 years.

A: SII for all-cause mortality; B: SIRI for all-cause mortality; C: SII for cardiovascular mortality; D: SIRI for cardiovascular mortality.

Abbreviations: *ROC* receiver operating characteristic, *SII* systemic immune-inflammation index, *SIRI* systemic inflammation response index


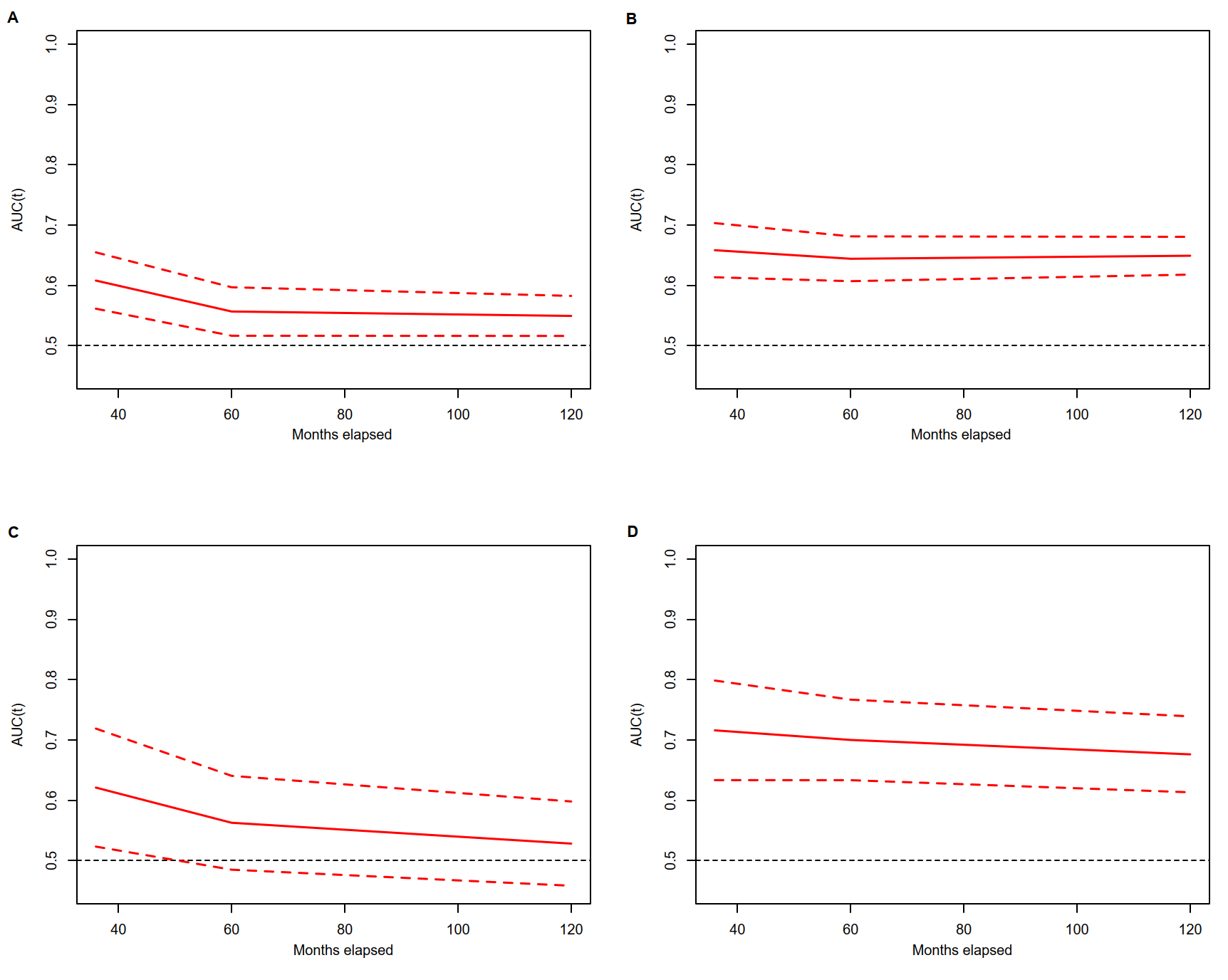


## Fig. S4 The treads of area under the ROC curve (with 95% confidence band) for predicting survival at 3, 5, and 10 years.

A: SII for all-cause mortality; B: SIRI for all-cause mortality; C: SII for cardiovascular mortality; D: SIRI for cardiovascular mortality.

Abbreviations: *AUC* area under the curve, *SII* systemic immune-inflammation index, *SIRI* systemic inflammation response index
